# Nanoparticles Based on Chondroitin Sulfate from Tuna Heads and Chitooligosaccharides for Enhanced Water Solubility and Sustained Release of Curcumin

**DOI:** 10.3390/polym15040834

**Published:** 2023-02-08

**Authors:** Yaowapha Waiprib, Pattarachat Ingrungruengluet, Wanchai Worawattanamateekul

**Affiliations:** 1 Department of Fishery Products, Faculty of Fisheries, Kasetsart University, Bangkok 10900, Thailand; 2 Center for Advanced Studies for Agriculture and Food (CASAF), Kasetsart University Institute for Advanced Studies, Kasetsart University, Bangkok 10900, Thailand

**Keywords:** chondroitin sulfate, tuna head, skipjack tuna (*Katsuwonus pelamis*), yellowfin tuna (*Thunnus albacares*), chitooligosaccharides, nanoparticles, curcumin, control release

## Abstract

This study aimed to separate chondroitin sulfate (CS) from the heads of skipjack tuna (*Katsuwonus pelamis*) and yellowfin tuna (*Thunnus albacares*), by-products derived from canned tuna processing, via a biological process. The use of 1% *w*/*w* papain and an incubation time of 48 h resulted in a degree of hydrolysis of 93.75 ± 2.94% and a CS content of 59.53 ± 1.77 mg/100 g. The FTIR spectra of extracted CS products exhibited identical functional groups found in commercially available CS. The molecular weights of CS extracted from skipjack and yellowfin tuna heads were 11.0 kDa and 7.7 kDa, respectively. Subsequently, a CH:CS ratio of 3:2 for CS and chitooligosaccharides (CH) was chosen as the optimal ratio for the preparation of spherical nanoparticles, with %EE, mean particle size, PDI, and zeta potential values of 50.89 ± 0.66%, 128.90 ± 3.29 nm, 0.27 ± 0.04, and −12.47 ± 2.06, respectively. The CU content was enhanced to 127.21 ± 1.66 μg/mL. The release of CU from this particular nanosystem involved mainly a drug diffusion mechanism, with a burst release in the first 3 h followed by a sustained release of CU over 24 h. The DPPH and ABTS scavenging activity results confirmed the efficient encapsulation of CU into CHCS nanoparticles. This study will provide a theoretical basis for CS derived from tuna head cartilages to be used as a functional component with specific functional properties in food and biomedical applications.

## 1. Introduction

Glycosaminoglycans (GAGs) are linear, negatively charged polysaccharides formed by covalently linked disaccharide units. Chondroitin sulfate (CS) is a sulfated GAG whose formulation displays a variation in molecular structure depending on the source of materials and separation methods [1,2,3], resulting in distinct biological properties such as antioxidant, anti-inflammatory, antitumor, anticoagulant, and antithrombogenic activities, and anti-viral and anti-metastatic properties [2,4,5]. Recently, fish by-product cartilages have gained interest in the production of CS, which traditionally uses cartilage from mammals and poultry, due to their lower environmental impact and health risk [1,3,4,5,6,7,8]. 

Thailand has established a large tuna-processing industry, playing a key role in international tuna trade as the world’s number one exporter of prepared or preserved tuna [9,10]. Within tuna and tuna-like species catches, skipjack (*Katsuwonus pelamis*) and yellowfin tuna (*Thunnus albacares*) account for over 55 percent of catches [10]. Among those economically important tuna species imported, skipjack tuna and yellowfin tuna were the first and second highest in volume imported to Thailand, respectively [11]. In general, fish by-products can account for up to 70% [12], 75% [13], 20–80% [14], and 50–70% [15] of the catch depending on species, postharvest, and the level of processing [13,14]. The majority of by-product components were contributed by the head fraction [12,16]. To date, despite some available information on the CS extracted from the head cartilage of edible bony fishes such as sturgeon and salmon have been revealed, showing that the head cartilage of bony fishes is a promising source for the preparation of CS [3,4,6], this information is still lacking for the CS extracted from the head cartilage of some commercially valuable tunas.

Curcumin (CU) is a natural polyphenol isolated from the rhizome of turmeric (*Curcuma longa* L.) [17]. CU research has attracted considerable interest in recent years due to its therapeutic potential, which includes but is not limited to being antioxidant, anti-inflammatory, anticarcinogenic, antimicrobial, hypoglycemic, neuroprotective, and hepatoprotective, and the mechanisms underlying therapeutic effects in diverse diseases and pathological states of cells and organisms have been well established [17,18,19,20,21]. CU is a substance that is generally recognized as safe, according to the Food and Drug Administration [22]. However, CU has limitations such as poor bioavailability, poor water solubility (1.34 μg/mL, 2.677 μg/mL [23], 6.79 μg/mL [24], <8 μg/mL [25], and 11 μg/mL [26]), rapid metabolism, and low absorption [24,25]. As a result, numerous strategies have been proposed, such as structural modification and the use of drug-delivery systems [19,20,27,28]. Effective methods for enhancing CU bioavailability recently include the use of adjuvants [20], CU-loaded nanostructured lipid carriers (NLCs) [29], certain CU nanoformulations, CU combination therapy [30], CU-loaded solid self-nanoemulsifying delivery systems (S-SNEDDS) [31,32], and CU-loaded biomimetic nanomedicines [33] due to advances in drug-delivery technology.

In recent years, polysaccharides have attracted a lot of attention as not only polymers for drug delivery but also therapeutics because of their versatility and distinct characteristics, including biocompatibility, biodegradability, low toxicity, and easy alteration and modification [34,35,36,37]. CS has a wide range of applications in the pharmaceutical, cosmetic, and food industries as a favorable biomaterial that has been applied extensively in the fields of drug delivery and tissue engineering due to its biocompatibility, non-toxicity, non-immunogenicity, and easy synthesis properties [4,38,39,40]. It is known that CS cannot produce a prolonged drug-release profile due to its water solubility [39,40,41,42]. CS-based nanocarriers for drug delivery have been reported in terms of the self-assembly of hydrophobically modified CS, CS-decorated nanocarriers, and some other CS-based nanocarriers [41,42,43]. Chitosan is a naturally occurring cationic polysaccharide that can readily interact with negatively charged polymers such as CS [42,43,44,45,46,47,48]. Chitooligosaccharide (CH) is the hydrolysis product of chitosan with less than 20 degrees of polymerization and an average molecular weight of less than 3.9 kDa [49,50]. While chitosan has been regarded as more popular in nano-carrier preparation due to its high molecular weight and viscosity [43,51,52,53], CH has been considered to possess higher bioactive activities such as antioxidant, anti-inflammatory, and immunomodulatory activities due to its water solubility and absorbability [49,54,55]. As a result, CH has gained much attention for its novel applications in nanoformulation to enhance the water solubility of poorly water-soluble drugs [54,55,56,57]. CS copolymer with chitosan has been proven as an efficient delivery system for curcumin (CU) [44,45]; however as far as we know, no previous research has investigated its copolymer with CH.

The objectives of this study were to separate CS from the tuna head cartilages of skipjack and yellowfin tuna and to use it as a copolymer with CH to enhance water solubility and to sustain the release of CU. At the same time, the antioxidant activities of the nanoparticles were evaluated. This study will provide a theoretical basis for CS derived from tuna head cartilage, a by-product derived from tuna processing, to be used as a functional component with specific functional properties in food and biomedical applications. 

## 2. Materials and Methods

### 2.1. Materials

Chondroitin sulfate A sodium salt from bovine trachea (CSA) (C9819, Sigma-Aldrich, Merck KGaA, Darmstadt, Germany) and chondroitin sulfate C sodium salt (CSC) (C433378, Toronto Research Chemicals Inc., North York, ON, Canada) were used as standards for chondroitin-4-sulfate and chondroitin-6-sulfate, respectively. Papain (Batch no.21012005, 3020 USP u/mg) was purchased from Shaanxi Pioneer Biotech Co., Ltd., Xian, China). Chitooligosaccharides (CH) (MW 2 kDa, >90% Deacetylation degree) was obtained from Qingdao Hehai Biotech Co., Ltd., Shandong, China. Curcumin (B21573) and tocopherol (A17039.18) were purchased from Alfa Aesar, Thermo Fisher Scientific, Heysham, Lancashire, UK. Pluronic F68 (TC222), and L-cysteine hydrochloride monohydrate (RM046) were obtained from HiMedia Laboratories Pvt. Ltd., Mumbai, India. Trichloroacetic Acid (06356) and ethylene diamine tetra acetic acid (03728) were obtained from Loba Chemie Pvt. Ltd., Mumbai, India. Ascorbic acid (KA79), boric acid (KA101), di-sodium hydrogen phosphate (KA621), potassium thiocyanate (KA421), potassium ferricyanide (KA393), sodium azide (KA1222), sodium di-hydrogen phosphate (KA471), sodium chloride (KA465), and hydrochloric acid (KA1367) were purchased from KemAus, Sydney, NSW, Australia. 1,9-dimethylmethylene blue zinc chloride double salt (341088), 2,2-diphenyl-1-picrylhydrazyl (DPPH) (D9132), 2,2′-azino-bis (3-ethylbenzothiazoline-6-sulfonic acid) (ABTS) (A1888), cetylpyridinium chloride (C0732), and the selenium reagent (1.08030.1000) were purchased from Sigma-Aldrich, Merck KGaA, Darmstadt, Germany. Potassium persulphate (121525.1210) and iron (III) chloride 6-hydrate (141358.1210) were supplied by PanReac AppliChem ITW Reagents, Darmstadt, Germany. Ethanol (E7025-1-2501, 99.99%) and sulphuric acid (S7064) were obtained from QRëC, Auckland, New Zealand. Methanol (LP230) was purchased from Honeywell Burdick & Jackson, Muskegon, MI, USA.

### 2.2. Measurement of Fish Length and Quantification of Components of Tuna-Processing By-Products

The skipjack and yellowfin tuna samples were kindly provided by the local tuna processing plant in Samut Sakhon, Thailand, and kept at −20 °C prior to use. Total length, standard length, head length, and depth of the fish body were measured, and the components of tuna processing by-products were quantified by percentage of total fish weight. The percentage yields of components were assessed using the following Equation (1):

%yield = (Component weight/Total fish weight) × 100(1)

### 2.3. Optimization of Enzymatic Extraction of Chondroitin Sulfate from Tuna Heads

#### 2.3.1. Sample Preparation

Tuna heads were provided by the local tuna processing plant in Samut Sakhon, Thailand, and kept at −20 °C prior to use. The head samples were prepared by the modified method previously described [7]. Briefly, the thawed samples were boiled in hot water at 90 °C for 15 min, and the muscle was manually discarded. The remaining were then minced to uniformity using a disperser (T25 digital Ultra-Turrax; IKA; Staufen, Germany), centrifuged at 15,000× *g* for 20 min at 4 °C (Suprema 21, Tomy Seiko Co., Ltd., Tokyo, Japan), and stored at −20 °C until use. The proximate composition analysis of the samples for moisture, protein, fat, and ash contents was conducted following the official methods of analysis of the Association of Official Analytical Chemists (AOAC) no. 931.04, 981.10, 922.06 and 920.153, respectively [58]. The total protein content was calculated by multiplying the nitrogen content by a conversion factor of 6.25 [59]. All analyses were conducted in triplicates. 

#### 2.3.2. Optimization of Enzymatic Extraction of CS from Tuna Heads

Enzymatic extraction of CS was carried out according to the modified method previously described [60]. The samples (20 g) were hydrolyzed by the papain enzyme at concentrations of 0.125, 0.25, 0.5, and 1% *w*/*w* in a 200 mL solution of 0.1 M sodium phosphate buffer pH 7.0 containing 0.005 M ethylenediaminetetraacetic acid, 0.02% sodium azide, and 0.005 M cysteine hydrochloride. Enzyme hydrolysis was carried out at 65 °C for 1, 2, 4, 8, 12, 24, 36, 48, and 60 h. Trichloroacetic acid was added to obtain the final concentration of 7% (*w*/*v*). The mixture was kept overnight at 4 °C and centrifuged at 15,000× *g* for 30 min at 4 °C to remove the precipitated protein. The supernatant was further dialyzed (MWCO 12,000-14,000, LA395, Dialysis Membrane-110, HiMedia Laboratories Pvt. Ltd., Mumbai, India) in chilled water for 24 h. The CS content was quantified by the method described previously with some modifications [61] using CSC as a standard. 1,9-dimethylmethylene blue was reacted with sulfated GAG, and the absorbance at 525 nm was measured using a spectrophotometer (SPECTROstar^®^ Nano, BMG LABTECH, Ortenberg, Germany). 

The degree of hydrolysis (DH) of hydrolysates was calculated from the ratio of the soluble protein in trichloroacetic acid to total protein after hydrolysis according to the modified method described previously [62]. The sample was mixed with 20% trichloroacetic acid at 1:1 ratio and stirred for 15 min. The mixture was then centrifuged at 7500× *g* for 15 min at 4 °C. The protein content was determined by AOAC no. 981.10 [58], and the conversion factor used was 6.25. The degree of hydrolysis (%DH) of hydrolysates was assessed using the following Equation (2):

% DH = Ps/Pt × 100(2)

where Ps and Pt are the amounts of protein dissolved in hydrolysate and the total amount of protein in the hydrolysate, respectively. 

The extracted CS samples were characterized by Fourier transform infrared (FTIR) spectroscopy over the range of 400–4000 cm^−1^ (Spectrum Two™ FTIR, PerkinElmer Inc., Waltham, MA, USA). Gel permeation chromatography (GPC) was used to determine the molecular weight of prepared CS with an ultrahydrogel linear column (10 µm, 7.8 mm × 300 mm, 1K–7M, Waters Corporation, MA, USA) and a refractive index detector (Waters 2414, Waters Corporation, MA, USA). Elution was performed using a 0.05 M sodium bicarbonate buffer (pH 11) as the mobile phase. The samples (2 mg/mL) were dissolved in eluent and filtered before injection with a 20 μL injection volume. The detector and column were warmed to 30 °C, and the flow rate was set to 0.6 mL/min. Pullulan standards (MW 5900–708,000 Da) were used for column calibration.

### 2.4. Preparation and Characterization of Curcumin-Loaded Chondroitin Sulfate–Chitooligosaccharide (CHCSCU) Nanoparticles

#### 2.4.1. Preparation of CU-Loaded Nanoparticles 

The CHCSCU nanoparticles were prepared according to the modified method previously described [43,63]. Firstly, the CU micelle was obtained by adding ethanolic solution of CU (1 mg/mL) dropwise to Pluronic solution (5 mg/mL) while being continuously stirred at 400 rpm (C-MAG HS7 Control, IKA^®^, Staufen, Germany) for 24 h at an equal volume. The CU micelle suspension was vacuum evaporated at 37 °C (Rotavapor R-124, Büchi Labortechnik AG, Flawil, Switzerland) and further slowly added at an equal volume into CH solution (1 mg/mL) containing CS (1 mg/mL) at weight ratios of 5:0, 4:1, 3:2, 2:3, and 1:4. The nanoparticles were formed under constant magnetic stirring at 400 rpm for 40 min at 25 °C and were further sonicated at 40 kHz, 100 W for 5 min in an ultrasound bath (DK-3000TS, DK-sonic, China). After sonication, the samples were centrifuged at 3000× *g* for 15 min at 4 °C and freeze dried (Scanvac Coolsafe Touch 95-15, LaboGene Aps, Lynge, Denmark). 

#### 2.4.2. Characterization of CU-Loaded Nanoparticles

The CU content was determined using a UV-VIS spectrophotometer (UV-1900i, Shimadzu Corporation, Kyoto, Japan). A spectral scan between wavelengths 370 nm and 460 nm produced a maximum absorption plateau between wavelengths 425 nm and 431 nm, and a wavelength of 427 nm was selected for the CU content determinations. The percentages of encapsulation efficiency (%EE) and loading capacity (%LC) were calculated by the following Equations (3) and (4).

%EE = Encapsulated curcumin/Total curcumin added × 100(3)

%LC = Encapsulated curcumin/Total nanoparticle weight × 100(4)

The particles size, polydispersity index (PDI), and zeta potential of nanoparticle samples were measured at 25 ± 0.5 °C by the dynamic light scattering (DLS) technique using a Zetasizer (Nano-ZS, Malvern Panalytical Ltd., Malvern, Worcestershire, UK) [64]. 

The nanoparticles and forming materials were characterized by FTIR and the morphology of nanoparticle was observed by field emission scanning electron microscopy (JSM-7600F Schottky Field Emission Scanning Electron Microscope, JEOL Ltd., Akishima, Tokyo, JAPAN).

#### 2.4.3. In Vitro Release Profiles

The in vitro release profiles of nanoparticles were examined using the dialysis bag method according to previously described method with some modifications [65]. Each sample (equivalent to 50 mg CU) was placed in a dialysis bag (MWCO 3500, Cellu Sep^®^CB-5015-46, Membrane Filtration products, Inc., Seguin, TX, USA) and immersed into 250 mL phosphate-buffered saline (PBS), pH 6.8, with constant shaking at 150 rpm at 37 ± 0.5 °C. At the specified time intervals, aliquots of the release medium (2 mL) were removed and replaced with fresh medium. The samples were analyzed for CU content as described previously in Section 2.4.2.

To analyze the release kinetics and mechanisms, data were fitted to the four mathematical models previously described [66,67] in the following Equations (5)–(8). $M_{t}$ is the fraction of CU released at time t.

(5) $$M_{t}=kt$$

where k is the zero order model constant.

(6) $$M_{t}=kt^{0.5}$$

where k is the Higuchi model constant.

(7) $$M_{t}=at^{0.5}+bt\;$$

where a and b are the diffusion rate and the erosion rate constants.

(8) $$M_{t}=kt^{n}$$

where k is the Korsmeyer–Peppas constant and n is a release mechanism constant. 

#### 2.4.4. Determination of Antioxidant Activities

The 2,2-diphenyl-1-picrylhydrazyl (DPPH) radical scavenging activity of nanoparticles was determined according to the method described previously with slight modifications [68]; 0.1 mM DPPH (in ethanol solution) and the sample (1:1 *v*/*v*) were reacted at room temperature for 30 min. The absorbance of the mixture was measured at 517 nm by a microplate reader. Ascorbic acid was used as the positive control. Each measurement was performed in triplicate, and the percentage of scavenging activity was calculated using Equation (9):

%DPPH radical scavenging activity = (Ac − As)/Ac × 100(9)

where Ac and As indicate the absorbance of the reaction mixture without a sample and with a sample, respectively. 

The 2,2′-azino-bis (3-ethylbenzothiazoline)-6-sulfonic acid) (ABTS) radical scavenging activity of nanoparticles was determined according to the method described previously with slight modifications [69]. Potassium persulfate (5.2 mM) was added to ABTS (14.8 mM) and incubated in the dark at room temperature for 16 h to obtain the ABTS radical solution. The solution was diluted to an absorbance of 0.70 ± 0.02 at 734 nm, and 100 μL of the ABTS radical solution was reacted with 100 μL of the sample for 10 min in the dark at room temperature, followed by measurement of the absorbance at 734 nm using the microplate reader. Tocopherol was used as the positive control. Each measurement was carried out in triplicate, and the percentage of scavenging activity was calculated using Equation (10):

%ABTS radical scavenging activity = (Ac − As)/Ac × 100(10)

where Ac and As indicate the absorbance of the reaction mixture without a sample and with a sample, respectively. 

### 2.5. Statistical Analysis

One-way analysis of variance (ANOVA) were conducted, followed by Duncan′s multiple range test for mean comparison. Pearson′s correlation coefficients (r) was used to determine the statistical relationship between two variables. The independent samples t-test was used to compare the means of two independent groups. All data are expressed as means ± standard deviation (SD) from three replicates.

## 3. Results and Discussion

### 3.1. Measurement of Fish Length and Quantification of Components of Tuna-Processing By-Products

**Figure 1:**
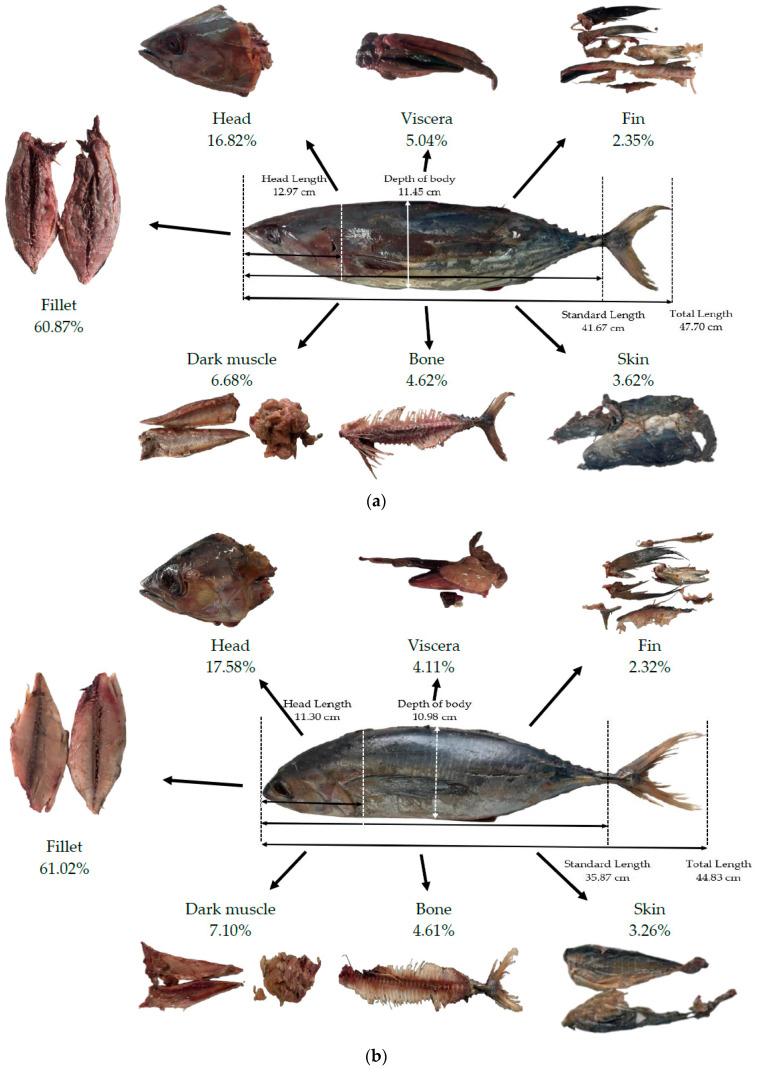
Fish length measurement (total length, standard length, head length, and depth of body) and components of tuna processing by-products by percentage of total fish weight: (**a**) skipjack tuna (*Katsuwonus pelamis*); (**b**) yellowfin tuna (*Thunnus albacares*).

As illustrated in Figure 1a,b, the total lengths of skipjack and yellowfin tuna measured in this study were 47.70 ± 1.95 cm, and 44.83 ± 1.95 cm, respectively. The results of this analysis were similar to the size of commonly captured skipjack tuna, which ranged from 40 to 80 cm; however, it was slightly smaller than the size of commonly captured yellowfin tuna and ranged from 60 to 150 cm [70]. As shown in Figure 1, the solid waste accounted for 39.13 ± 9.77% and 38.98 ± 6.93% of total weight for skipjack and yellowfin tuna, respectively. The solid waste generated by the tuna processing could be as high as 50–70% of the original material [15]. However, the tuna meat fractions, 60.87 ± 9.77% and 61.02 ± 6.93% of total weight for skipjack and yellowfin tuna, respectively, were consistent with previous findings that tuna meat made up 62% of the total fish composition [15,70]. As previously reported, the head fraction varied greatly among different types of fish [12]. The tuna head residues accounted for 16.82 ± 1.07% and 17.58 ± 1.97% of total weight, equivalent to 42.98 ± 2.73% and 45.11 ± 4.60% of total solid waste, for skipjack and yellowfin tuna, respectively. The result from this present study showed similar findings suggesting that the fish head fraction contributed to the major portion of by-product components such as Alaska red salmon (*Oncorhynchus nerka*) [16], meagre sea beam (17.09–18.74%), and gilthead sea bream (16.70–18.49%) [12].

As shown in Table 1, the protein and ash contents were significantly different between skipjack and yellowfin tuna heads (*p* < 0.05), whereas the moisture and lipid contents were not significantly different (*p* < 0.05). The moisture contents of tuna heads in this study ranged from 65.09 to 66.70 g/100 g fresh weight, which was consistent line with previous findings of high moisture content in skipjack tuna head (75.6 ± 0.5%) [71], rainbow trout head (62.4 ± 0.7%) [71], meagre head (64–68.9%), and gilthead sea bream head (57.3–62.4%) [12]. Protein content, lipid content, and ash content varied greatly among different species of fish heads [12,16,71,72]. Overall, these present findings on moisture, protein, and lipid contents were in accordance with previous findings [71,72], except for ash content. The high ash content may be associated with the presence of a high proportion of bone in fish heads [71,72]. 

### 3.2. Optimization of Enzymatic Extraction of Chondroitin Sulfate from Tuna Heads

**Figure 2:**
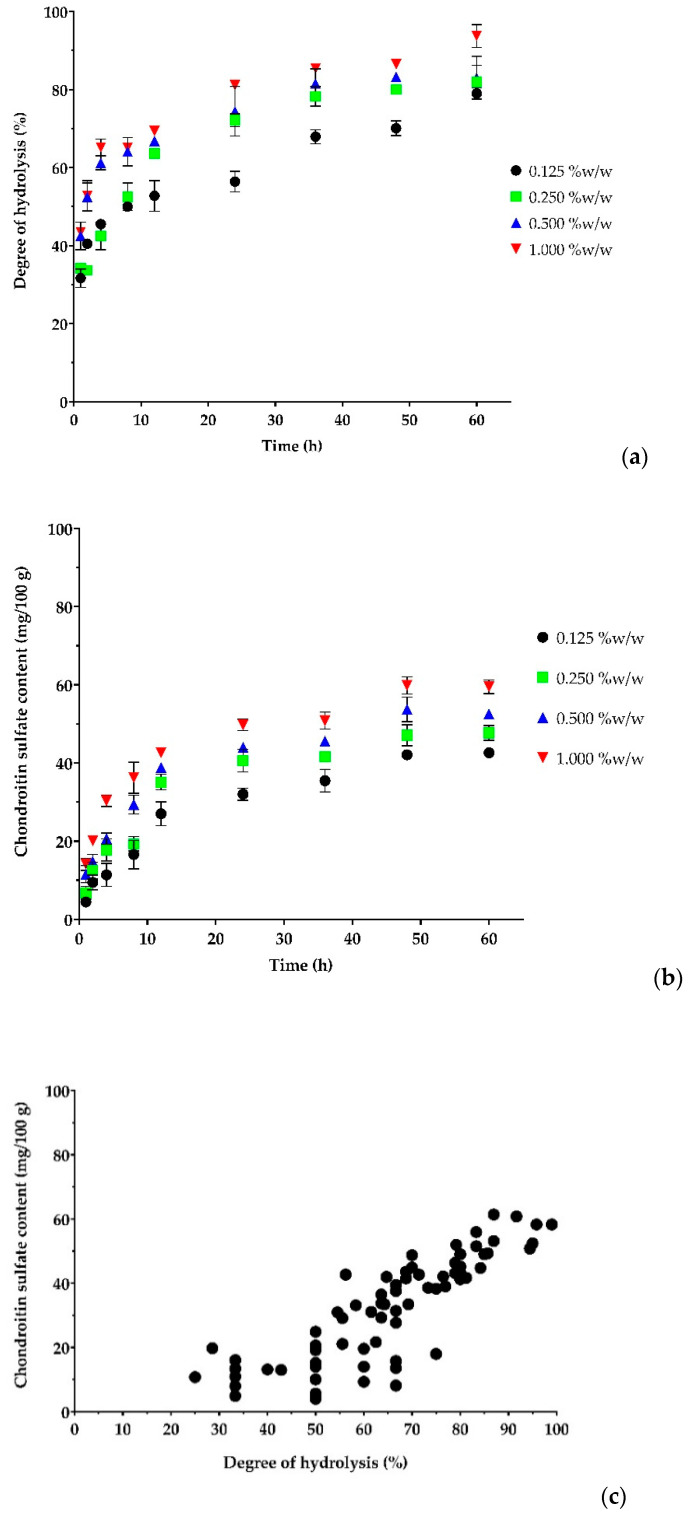
The effects of enzyme concentration and reaction time on (**a**) degree of hydrolysis; (**b**) chondroitin sulfate (CS) content; (**c**) Pearson’s correlation coefficient between the degree of hydrolysis and CS content (r = 0.844, *p* < 0.01).

Figure 2a,b demonstrated that the %DH and CS contents gradually increased with the progress in the reaction time and leveled off at about 48 h. When the reaction time increased from 1 h to 48 h, the %DH and CS contents were substantially increased; however, upon extending the incubation time beyond 48 h, no significant increase in the %DH and CS contents was observed. After 60 h, as the enzyme concentration increased from 0.125% to 1% *w*/*w*, the %DH increased from 79.00 ± 1.41% to 93.75 ± 2.94%and the CS content increased from 42.62 ± 0.93 mg/100 g to 59.53 ± 1.77 mg/100 g. The maximum %DH and CS contents were achieved by using a 1% *w*/*w* enzyme concentration within the time range of 48 h. This is in accordance with previous studies that found that the CS contents extracted from different kinds of fish and squid heads ranged from 8 to 109 mg/100 g of dry defatted tissue [73]. Previous studies have also shown that the CS contents extracted from codfish, tuna, salmon, spiny dogfish, and monkfish fish bones were 0.011%, 0.023%, 0.1% 0.28%, and 0.34% *w*/*w*, respectively [74]. As seen in Figure 2c, there was a positive correlation between %DH and CS content (correlation coefficient, r = 0.844, *p* < 0.01). These positive correlations between %DH and CS content could be explained by the fact that the longer incubation time and higher concentration of the enzyme used would allow the enzyme to react on a protein to a greater extent, resulting in an elevation of %DH and a liberation of CS. The concentration of enzyme used and incubation time significantly influence hydrolysis efficiency and CS yield. The papain enzyme used in this study was one of the most often employed enzymes that has been proven for its ability to release CS from different kinds of fish raw materials [1,4]. Based on the obtained results, an enzyme concentration of 1% *w*/*w* and a reaction time of 48 h were chosen as the optimal enzyme concentration and reaction time for CS separation. This result ties in well with a previous study wherein the clear solution was obtained after 48 h of hydrolysis at 65 °C with papain 0.4% *w*/*w* [60], as well as after 24 h of hydrolysis at 60 °C with papain 0.6% *w*/*w* [74]. 

**Figure 3:**
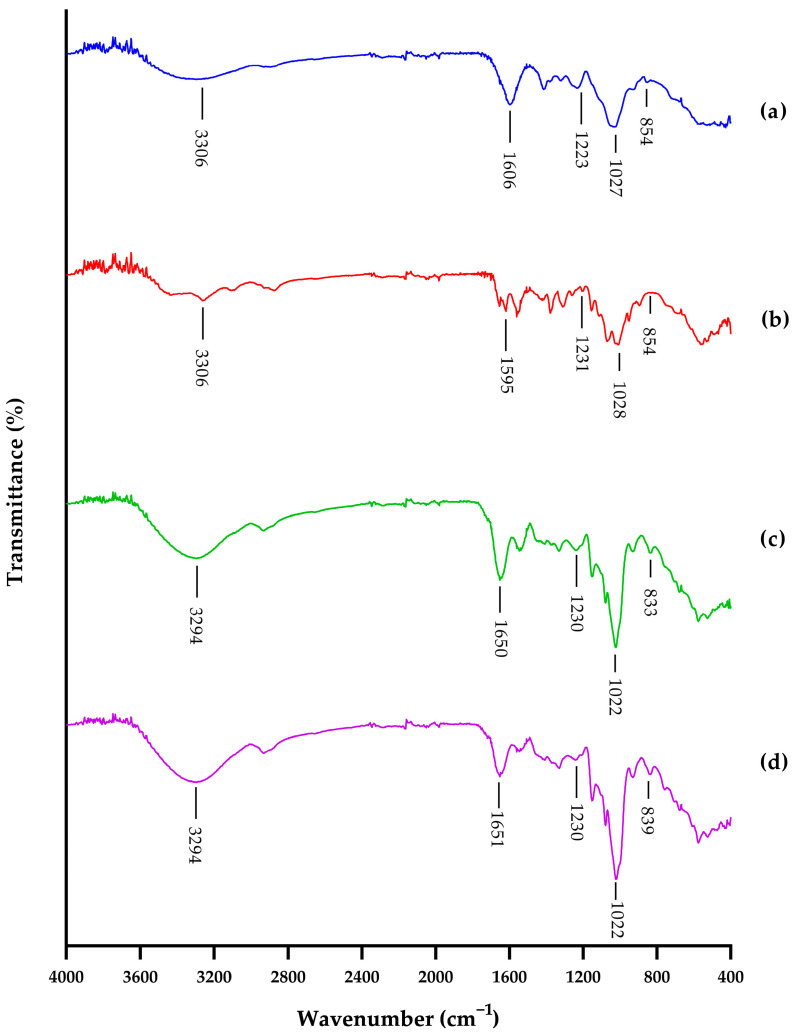
FTIR spectra of (**a**) chondroitin-4-sulfate; (**b**) chondroitin-6-sulfate; (**c**) chondroitin sulfate from head cartilage of skipjack tuna (*Katsuwonus pelamis*); (**d**) chondroitin sulfate from head cartilage of yellowfin tuna (*Thunnus albacares*).

As shown in Figure 3, the FTIR spectral data of the –CONH vibration of the amide group coupling of C–O stretching vibrations, S=O stretching vibrations, and C–O–S axial and equatorial bending vibrations prove that the isolated samples of skipjack and yellowfin tuna heads are from the CS product. The characteristic peaks of –CONH were observed at 1606, 1595, 1650, and 1651 cm^−1^ for standard CSA, CSC, and CS extracted from skipjack and yellowfin tuna heads, respectively. The results were in good agreement with previous work on extracted samples of buffalo tracheal, nasal, and joint cartilages [61]; crocodile hyoid, rib, sternum, and tracheal cartilages; shark fin; ray cartilage; chicken keel cartilage [60]; cephalopods [75]; yellowfin gill [76]; and Atlantic bluefin tuna (*Thunnus thynnus*) skins [77]. The characteristic peaks of S=O were observed at 1223, 1231, 1230, and 1230 cm^−1^ for standard CSA, CSC, and CS derived from skipjack and yellowfin tuna heads, respectively. The results were in line with previous studies on extracted samples of buffalo tracheal, nasal and joint cartilages [61]; crocodile hyoid, rib, sternum, and tracheal cartilages; shark fin; ray cartilage; chicken keel cartilage [60]; cephalopods [75]; and Atlantic bluefin tuna skins [77]. The characteristic peaks of C–O–S were observed at 854, 829, 833, and 839 cm^−1^ for standard CSA, CSC, and CS extracted from skipjack and yellowfin tuna heads, respectively. The results were in line with previous studies on extracted samples of buffalo tracheal, nasal, and joint cartilages [61]; crocodile hyoid, rib, sternum, and tracheal cartilages; shark fin; ray cartilage; chicken keel cartilage [60]; cephalopods [75]; Atlantic bluefin tuna skins [77]; and Nile tilapia (*Oreochromis niloticus*) bones [78]. The slight variation in this characteristic peak observed in this study indicated that they were made up of different proportions of CSC and CSA [60,61]. CS from cartilaginous fish is characterized by a high percentage of CSC, whereas that from terrestrial vertebrates contains a higher percentage of CSA [4,60,61,73,79,80].

The molecular weights of CS derived from skipjack and yellowfin tuna heads were determined to be 11.0 and 7.7 kDa, respectively. This is in accordance with previous studies that found the molecular weight of CS contents extracted from spiny dogfish, codfish, salmon, tuna, and monkfish bones to be 13.46 kDa, 18.12 kDa, 20.07 kDa, 32.94 kDa, and 48.68 kDa, respectively [74], and that of codfish (*Gadus macrocephalus*) bones to be 12.3 kDa [81]. In addition, the molecular weight of CSA and CSC used in this study were also determined to be 102.54 kDa and 70.64 kDa, respectively, by the same method, indicating that there is variation in the molecular weight of CS reported, ranging from 5 kDa to 100 kDa depending on the source and the tissue [1] and extraction method [78,82].

### 3.3. Effects of CH:CS Ratio on CU-Loaded Nanoparticle Characteristics

**Figure 4:**
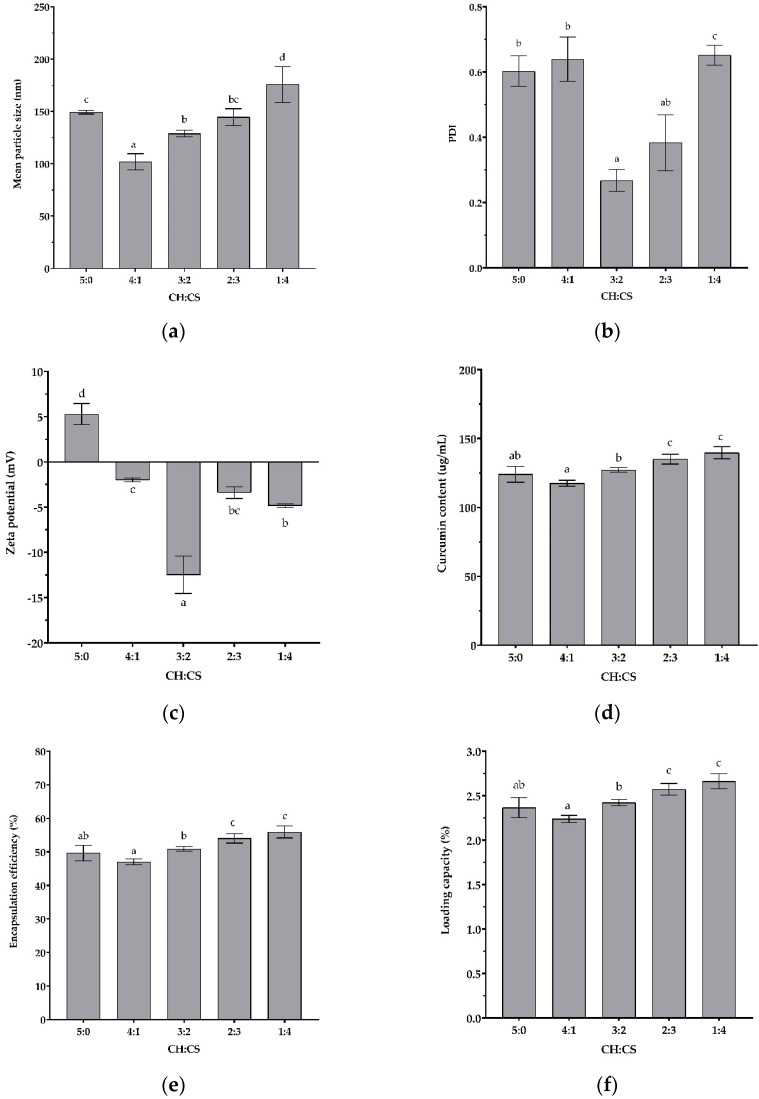
Effects of chitooligosaccharides: chondroitin sulfate (CH:CS) ratio on nanoparticle characteristics: (**a**) mean particle size; (**b**) polydispersity index (PDI); (**c**) zeta potential; (**d**) curcumin content; (**e**) encapsulation efficiency; (**f**) loading capacity. Values with different letters differ significantly (*p* < 0.05).

According to the results shown in Figure 4, CU-loaded nanoparticle characteristics were significantly dependent on the CH:CS ratio (*p* ˂ 0.05). As shown in Figure 4a–c, the mean particle size of all formulations was approximately below 200 nm, with variation in PDI and zeta potential values depending on the polymer weight ratio. The zeta potential value was positive for CU-loaded with CH without CS (CH:CS = 5:0), while the zeta potential values were negative for CU-loaded with both polymers. The –NH_2_ in CH was protonated to –NH_3_+, resulting in positive zeta potential values, while CS was deprotonated to form –OSO_3−_, resulting in negative zeta potential values on the surface of the nanoparticles [43]. The negative zeta potential was observed due to its lower amount of protonated CH amine groups, according to a previous study [44]. An increased CS content increased the number of free –OSO_3−_ groups on the surface of the polymer, resulting in a larger hydrodynamic diameter. The highest magnitude of zeta potential values (−12.47 ± 2.06) appeared when the CH:CS ratio was 3:2, corresponding to a PDI value below 0.3 (0.271 ± 0.04), which is considered acceptable for a monodisperse and homogenous population of nanoparticles in drug-delivery applications [68]. 

As shown in Figure 4d, the CU contents of nanoparticles prepared from two polymers were gradually enhanced with an increase in CS content (*p* ˂ 0.05). The extent of CU content enhancement in this study was comparable to a previous study on the shellac encapsulation of CU [83]. While there was no significant difference between CU contents between nanoparticles prepared from CH:CS at 5:0 and CH:CS at 3:2, there were significant differences between the mean particle size, PDI, and zeta potential values described above (*p* ˂ 0.05). The present nanosystem has shown excellent potential to improve the water solubility of CU. The enhanced water solubility could be caused by the smoother surface of CU-loaded nanoparticles and the decreased characteristic morphology of pure CU, which was in good agreement with the previous findings for the naringin–CH complex [54]. 

Similar patterns in the results were demonstrated in Figure 4e and Figure 4f for %EE and %LC, respectively. The %EE of the CU-loaded nanoparticles in this study ranged from 49.66 ± 2.33% to 55.94 ± 1.76% depending on the polymer weight ratio, which is consistent with previous findings on CU loaded in the chitosan and CS system, which showed a %EE ranging from 62.4 to 63.8% depending on the pH of the chitosan solution and the chitosan and CS weight ratio [44,48]. The %EE of the proanthocyanidin-loaded chitosan–CS system was also reported to be in a range of 17–56% affected by the chitosan and CS weight ratio, and drug concentration [43]. The %LC of the CU-loaded nanoparticles in this present study was in a range of 2.36 ± 0.11% to 2.66 ± 0.08%, depending on the polymer weight ratio. As mentioned above, when the ratio of CS increased, the negatively charged particles increased, which could lead to more electrostatically bound sites for CH, resulting in an increase in the mean particle size. However, the molecular weight of CS (11 kDa) was much higher than that of CH (2 kDa); thus, the increase in the mean particle size when the CH:CS ratio exceeded 3:2 (*w*/*w*) could be due to the entanglement of CS chains, leading to more coacervation and complexation [43]. The increased number of electrostatically interacting sites increased the amount of nanoparticles, making it easier for the nanoparticles to embed CU [84], resulting in higher %EE and %LC, as shown in Figure 4e,f. Based on the obtained results, the CH:CS ratio of 3:2 was chosen as the optimal ratio for nanoparticle preparation according to the low PDI and high magnitude of zeta potential values, resulting in a greater stability of CU nanoparticles [45]. The current study confirmed the findings about the CH’s ability to enhance the water solubility of poorly water-soluble drugs such as hesperidin [55], rutin [56], naringin [54], β-carotene [57], and silymarin [85], as well as CS that could form polyelectrolyte complexes through an electrostatic interaction with positively charged CH, thus providing an optimal strategy to maintain CS in the solid state for use as a drug-delivery system [45]. 

### 3.4. Characterization of Curcumin-Loaded Chitooligosaccharide–Chondroitin Sulfate (CHCSCU) Nanoparticles

#### 3.4.1. FTIR Spectra

**Figure 5:**
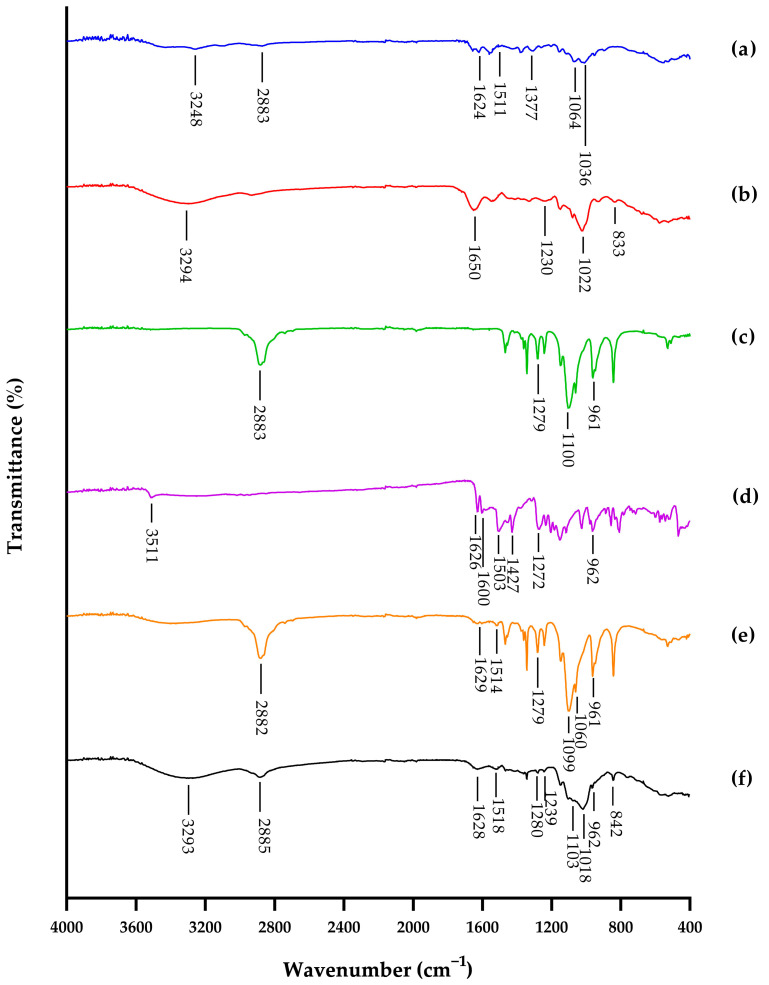
FTIR spectra of (**a**) chitooligosaccharide (CH); (**b**) chondroitin sulfate (CS) extracted from head cartilage of skipjack tuna (*Katsuwonus pelamis*); (**c**) Pluronic; (**d**) curcumin (CU); (**e**) CHCU nanoparticles; (**f**) CHCSCU nanoparticles.

FTIR spectra present the main peaks of the functional groups present in the nanoparticles and their possible interactions (Figure 5). As shown in Figure 5f, a band appeared at 1018 cm^−1^ in the spectrum of the CHCSCU nanoparticles assigned to NH_3_+ and SO_3_− stretching, indicating evidence of the interaction between both polymers according to previous findings (1020 cm^−1^) [44,45]. The FTIR spectra also confirmed the presence of CS in CHCSCU nanoparticles, with peaks observed around 1239 cm^−1^ and 842 cm^−1^, respectively, assigned to the S=O and C-O-S bonds (1238–1060 cm^−1^ and 856 cm^−1^) [44,45]. 

As presented in Figure 5e,f, the FTIR spectra confirmed the incorporation of Pluronic in the CHCU and CHCSCU nanoparticles. The peaks observed in the CHCU and CHCSCU spectra at around 2882 cm^−1^ and 2885 cm^−1^, respectively, were assigned to C-H bond stretching and those at 1099 cm^−1^ and 1103 cm^−1^ were assigned to C-O bond stretching according to previous findings (2881 cm^−1^ and 1108 cm^−1^) [86]. The FTIR spectra also confirmed the encapsulation of CU in the CHCU and CHCSCU nanoparticles, with peaks around 961 cm^−1^, and 962 cm^−1^, respectively, assigned to benzoate trans -CH in a previous study (960 cm^−1^) [26]. In addition, the peaks observed in the CHCU and CHCSCU spectra at around 1629 cm^−1^ and 1628 cm^−1^, respectively, were assigned to the C=O bond of the conjugated ketone according to previous findings (1627 cm^−1^) [44]. In brief, the FTIR spectra indicated that the CHCSCU and CHCU nanoparticles were successfully synthesized.

#### 3.4.2. Nanoparticle Morphology

**Figure 6:**
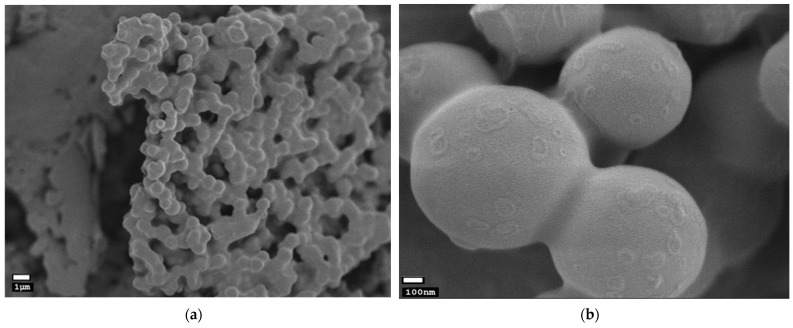
Field-emission scanning electron microscope images of curcumin loaded chitooligosaccharide–chondroitin sulfate-nanoparticles with scale bars of (**a**) 1 μm (×5000); and (**b**) 100 nm (×50,000).

Field-emission scanning electron microscope images of CHCSCU nanoparticles are presented in Figure 6a, with a scale bar of 1 um (×5000), and Figure 6b, with a scale bar of 100 nm (×50,000). As observed, the morphology of the produced particles is spherical, with a quite smooth surface and some aggregation tendency, in line with the previous studies related to chitosan–CS nanoparticle systems [44,45,48]. The particle size measured with a field-emission scanning electron microscope was found to be larger than the result obtained using dynamic light scattering. Among the several methods, dynamic light scattering approach is precise, dependable, repeatable, and suitable for nanoparticle size measurement [87]. This discrepancy could be attributed to the loss of stability of the nanoparticles during the freeze-drying process and their relative aggregation, while diluted samples were analyzed by the dynamic light scattering method, which prevented their aggregation [88]. Summer savory (*Satureja hortensis* L.) essential oil-loaded chitosan nanoparticles and β-carotene-CH complexes yielded similar result [57]. The enhanced water solubility could be caused by the smoother surface of CU-loaded nanoparticles and the decreased characteristic morphology of pure CU, which was in good agreement with the previous findings for the naringin–CH complex [54].

#### 3.4.3. In Vitro Release Profiles

**Figure 7:**
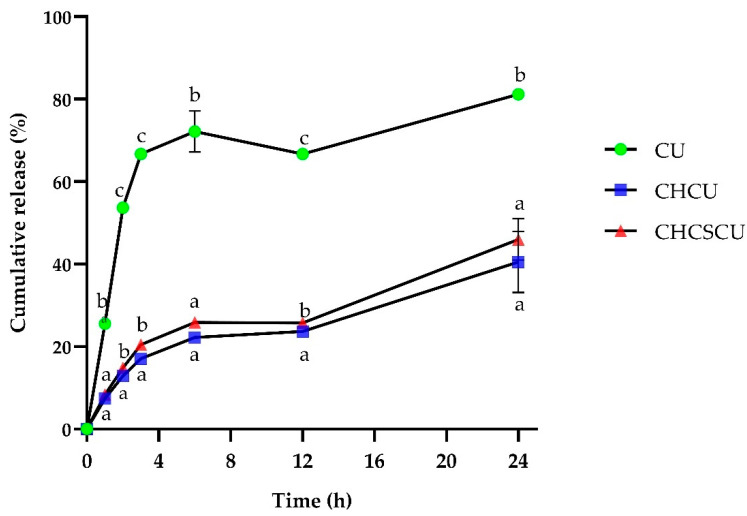
In vitro release profiles of curcumin (CU), CU-loaded nanoparticles with chitooligosaccharides (CH), and chondroitin sulfate (CS) in phosphate-buffered saline, pH 6.8. Notes: Mean ± SD, n = 3.

The in vitro release study showed sustained release of CU from CHCU and CHCSCU formulations compared with the free CU suspension, as illustrated in Figure 7. It revealed a burst release in the first 3 h, followed by a sustained release of CU over 24 h. In contrast, approximately 80% of the free CU was found in the release medium, indicating that CU displayed a rather fast release, that the dialysis bag had no detention on drug release after 24 h, and that the CHCU and CHCSCU nanoparticles released less than 20% of their CU content after 3 h and approximately 40% after 24 h. This is in line with previous studies showing that about 20–40% of CU contents were released from nanoparticles, while about 80% of CU contents were released from free CU in the medium studied within 24 h [67,89,90]. The results suggest that CU molecules were well-encapsulated within the nanoparticles, hence enhancing CU stability, and were released in a controlled manner, hence providing sustained delivery.

The model parameters after fitting the in vitro drug release data to four different mathematical models are presented in Table 2. The results indicate that the release data of nanoparticles were adequately fitted to the Higuchi model, Kopcha, and Korsmeyer–Peppas models, with R^2^ values higher than 0.9471. The value of n as a release exponent in the Korsmeyer–Peppas model was calculated to characterize the release mechanism. The values of n determined for the CHCU and CHCSCU nanoparticles were 0.4610, and 0.4471, indicating both diffusion-controlled drug release and swelling-controlled drug release or a pseudo-Fickian diffusion mechanism [67,91]. However, the ratio of a/b in the Kopcha model was determined to be higher than 1, indicating that Fickian diffusion was the main release mechanism, in accordance with previous findings [66,67]. The obtained results from this study revealed that the release of CU from this particulate system involved mainly a drug diffusion mechanism, in line with previous results on CU-loaded [44,47], and protein-loaded chitosan and CS nanosystems [67]. It was verified that the CHCU and CHCSCU nanoparticles displayed a similar pattern of control release kinetics. The nanoparticles prepared in this study demonstrated a clear potential for use as drug carriers for controlled drug release.

#### 3.4.4. Antioxidant Activities

**Figure 8:**
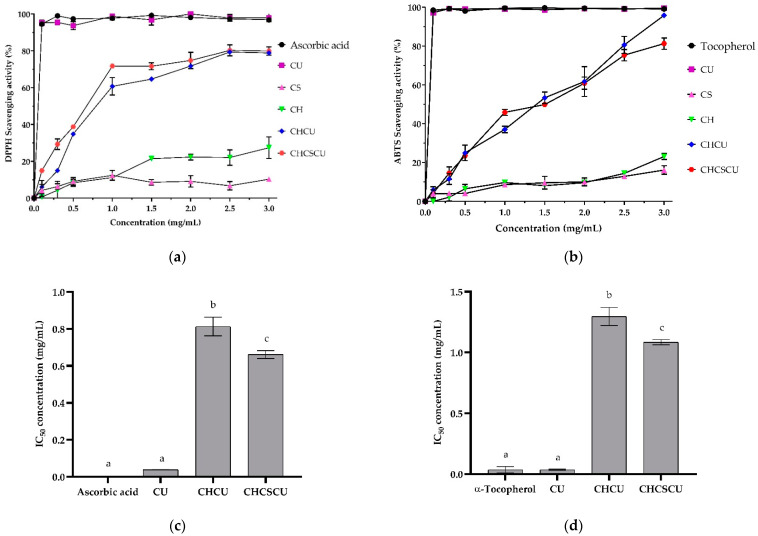
Antioxidant activities of curcumin (CU), chondroitin sulfate (CS) from head cartilage of skipjack tuna (*Katsuwonus pelamis*), chitooligosaccharides (CH), CU-loaded nanoparticles (CHCU, and CHCSCU): (**a**) 2,2-diphenyl-1-picrylhydrazyl (DPPH) radical scavenging activities, with ascorbic acid used as the positive control; (**b**) 2,2′-azino-bis (3-ethylbenzothiazoline)-6-sulfonic acid) (ABTS) radical scavenging activities, with α-Tocopherol used as the positive control; (**c**) the half maximal inhibitory concentration (IC_50_) (DPPH); (**d**) IC_50_ (ABTS). Values are expressed as means ± standard deviation (n = 3).

As shown in Figure 8a,b, the antioxidant activities of the CU-loaded nanoparticles (CHCU and CHCSCU) and their nanoformulation materials (CH and CS) increased with an increase in their concentrations. The CHCU and CHCSCU nanoparticles exhibited greatly increased antioxidant activities compared to the CS and CH nanoformulation materials, which also exhibited their own antioxidant effects as a benefit of being natural polysaccharides [34]. The DPPH radical scavenging activities of CS from the present study are in accordance with those of previous studies on the DPPH scavenging activities of CS derived from Nile tilapia bones, possessing 5–10% DPPH scavenging activities at their corresponding concentration of 0.2–1.0 mg/mL [78]. Previous studies have also shown that CS derived from bovine CS, shark CS, and Chinese giant salamander CS exhibited 16–26%, 22–32%, and 15–25% DPPH scavenging activities at their corresponding concentration of 0.4–2 mg/mL [92]. The DPPH and ABTS radical scavenging activities of CH were in agreement with previous reports that the antioxidant activities of CH greatly varied depending on its molecular structure such as its molecular weight, degree of deacetylation, and degree of polymerization [93]. Others have also shown that the antioxidant activity of CH has been found to be increased by the conjugation of antioxidant agents [54,55,56,57].

Single-electron transfer or hydrogen atom transfer reaction kinetics are the basis for the DPPH and ABTS radicals’ scavenging activities [94]. The discrete antioxidant activity presented by CHCU nanoparticles, prepared at the CH:CS ratio of 5:0, could be attributed to CH, whose their positive charges on the surface of the nanoparticles offer hydrogen to DPPH radicals [95]. In contrast, CHCSCU nanoparticles, prepared at a CH:CS ratio of 3:2, whose negative charged sulfate moieties are attached to the disaccharide units of their polysaccharide chains, could lead to a weaker dissociation energy of the hydrogen bonds between the polysaccharide chains, offering the donation of hydrogens from the hydroxyl groups [96]. This finding suggested that the environment for CU reactivity with DPPH and ABTS radicals was improved by encapsulating CU in polymeric nanocomposites, making it easier for CU to provide hydrogen atom transfer [94]. The IC_50_ values of CHCSCU nanoparticles on DPPH and ABTS scavenging activities were found to be significantly lower than those of CHCU nanoparticles, as illustrated in Figure 8c and Figure 8d, respectively. The fact that CHCSCU nanoparticles exhibited stronger scavenging activities than CHCU nanoparticles could be plausibly due to the fact that more CU molecules interacted with free radicals at the interface when CHCSCU nanoparticle possessed a larger specific surface area (Figure 4a) [97]. As a result, CHCSCU nanocomposites with a greater specific surface area may dissolve faster, resulting in a higher diffusion rate profile compared to CHCU nanoparticles (Figure 7). The antioxidant results confirm the efficient encapsulation of CU into CH and CHCS polymeric materials and suggest nanoparticles as a promising candidate to be used in food products.

## 4. Conclusions

The main conclusion that can be drawn is that tuna head cartilages could be utilized for CS production. The isolation of CS was primarily subjected to optimal protein elimination via hydrolysis with papain enzyme. The FTIR spectra of partially purified CS exhibited identical functional groups found in commercial CSA and CSC. The molecular weights of CS extracted from skipjack and yellowfin tuna head cartilages were determined to be 11.0 and 7.7 kDa, respectively. Subsequently, the present nanoparticles derived from CS extracted from tuna heads and CH have shown excellent potential to improve the water solubility of CU. The release of CU from this particular nanosystem involved mainly drug diffusion mechanisms, showing a burst release in the first 3 h, followed by a sustained release of CU over 24 h, suggesting that CU molecules were well-encapsulated within the nanoparticles and were released in a controlled manner. The antioxidant results confirmed the efficient encapsulation of CU into CHCS nanoparticles and suggested nanoparticles as a promising candidate to be used in food products. This study provided a theoretical basis for CS derived from tuna head cartilage to be used as a functional component with specific functional properties in food and biomedical applications. However, the empirical results presented here should be interpreted in light of the limitations raised by the structural and compositional analyses of CS extracted from a new source, as it is well known that the water solubility and biological properties of natural polysaccharides, such as antioxidant activities, vary greatly depending on their structural and composition characteristics. Future investigations on the structural and compositional analysis of CS are necessary to validate the kinds of conclusions that can be drawn from this study.

## Figures and Tables

**Table 1:** Proximate composition of skipjack heads and yellowfin tuna heads (g/100 g fresh weight) of this work compared with previous studies on skipjack tuna head [71] and yellowfin tuna head [72].

Parameters	Skipjack Tuna Heads		Yellowfin Tuna Heads	
	In the Present Study	Li et al. [71]	In the Present Study	Oliveira et al. [72]
Moisture	66.70 ± 0.70	75.6 ± 0.5	65.09 ± 1.31	70.1
Protein	18.35 ± 0.23 ^a^	18 ± 3	16.96 ± 0.33 ^b^	15.1
Lipid	3.64 ± 0.21	4.8 ± 0.5	4.12 ± 0.63	7.1
Ash	9.71 ± 0.22 ^a^	3.88 ± 0.08	8.05 ± 0.31 ^b^	5.18

Values in the same row followed by different lowercase superscript differ significantly (*p* < 0.05).

**Table 2:** Model parameters for curcumin (CU), and CU-loaded nanoparticles with chitooligosaccharides (CH), and chondroitin sulfate (CS) after fitting the in vitro drug release data to four different mathematical models.

	Zero Model		Higuchi Model		Kopcha Model			Korsmeyer–Peppas Model		
	k	R^2^	k	R^2^	a	b	R^2^	k	n	R^2^
CU	4.5645	0.4498	453.7787	0.7076	21.3021	0.0001	0.7076	3.17×10^7^	0.2171	0.8808
CHCU	1.9139	0.8707	66.9977	0.9658	8.1239	0.0002	0.9656	116.35	0.4610	0.9671
CHCSCU	2.1641	0.8486	84.9332	0.9471	9.2159	0.0011	0.9471	193.36	0.4471	0.9496

## Data Availability

The authors confirm that the data supporting the findings of this study are available within the article. Derived data supporting the findings of this study are available from the corresponding author [Y.W.] on request.
